# Exoscopic removal of the fourth ventricle choroid plexus papilloma with use of a midline suboccipital osteoplastic craniotomy

**DOI:** 10.3171/2023.10.FOCVID23106

**Published:** 2024-01-01

**Authors:** Rinat A. Sufianov, Ilshat A. Gaysin, Iurii A. Iakimov, Albert A. Sufianov

**Affiliations:** 1 Department of Neurosurgery, Sechenov First Moscow State Medical University (Sechenov University), Moscow, Russian Federation;; 2 Department of Pediatric Neurosurgery of Federal Center of Neurosurgery, Federal Center of Neurosurgery of Ministry of Health of the Russian Federation, Tyumen, Russian Federation;; 3 Educational and Scientific Institute of Neurosurgery, Peoples’ Friendship University of Russia (RUDN University), Moscow, Russian Federation; and; 4 King Edward Medical University, Lahore, Pakistan

**Keywords:** exoscope, choroid plexus papilloma, fourth ventricle, midline suboccipital osteoplastic craniotomy

## Abstract

Choroid plexus papillomas are relatively rare vascular tumors. In this video, the authors present a pediatric patient who underwent exoscopic removal of the fourth ventricle choroid plexus papilloma with the use of a midline suboccipital osteoplastic craniotomy. The exoscope in the fourth ventricle lesion helps to improve visualization in all directions, with the surgeon being able to maintain a comfortable position throughout the procedure. In addition, the midline suboccipital osteoplastic craniotomy helps to reduce the potential risks of complications, in particular, CSF leak and craniovertebral junction instability.

The video can be found here: https://stream.cadmore.media/r10.3171/2023.10.FOCVID23106

## Transcript

### 0:20 Patient History.

This video case report demonstrates an exoscopic removal of the fourth ventricle choroid plexus papilloma with use of a midline suboccipital osteoplastic craniotomy. An 18-year-old boy presented with 2-year-long moderate tension headache and 1-year-long tremor with gradual deterioration. Neurological examination revealed the presence of intention tremor, otherwise without features.

### 0:44 Preoperative Imaging.

Preoperative MRI demonstrated a cauliflower-like tumor completely occupying the fourth ventricle, with intensive and homogeneous contrast enhancement. The first line of treatment for all choroid plexus tumors is maximal surgical resection.^1^

### 0:59 Patient Positioning and Surgical Plan.

In this case, the patient was placed in Concorde position. The use of an exoscope in lesions of the fourth ventricle is associated with better visualization. A flat angle of view allows direct access to the tumor of the fourth ventricle practically through the foramen of Magendie, enlarged because of the tumor, which allows minimizing the size of the craniotomy and damage to the surrounding tissue.^2^ Due to the compact design and mobility of the exoscope, the surgeon can change the viewing angle in a wide range without changing the position of their body, and with less arm strain.^3,4^

### 1:35 Midline Suboccipital Osteoplastic Craniotomy.

Midline suboccipital osteoplastic craniotomy, also named as "cobra craniotomy," is a variant of craniotomy where the atlanto-occipital membrane is preserved. In the existing studies, it is presented as an alternative method potentially reducing the risk of postoperative complications.^5,6^

### 1:53 Operative Video.

A small straight incision was performed with dissection of the muscles along the midline. The occipital bone and C1 were skeletonized with preserving the atlanto-occipital membrane. One burr hole craniotomy was made followed by dissection of the atlanto-occipital membrane from the dura. Intraoperative ultrasound showed hyperechogenic tumor in the fourth ventricle. The dura was opened with a C-shaped incision. The first view revealed a choroid plexus tumor highly adhered to the surrounding structures. The tumor was dissected and devascularized stepwise. In this case of a vascular tumor, Thulium:YAG laser was used for inner decompression; then the tumor was collected for pathology study. The use of Thulium:YAG laser contributed to minimizing the bleeding from the tumor surface during its removal. The exoscope was helpful to easily visualize the roof of the fourth ventricle and remove the remnants of the tumor. The arachnoid membrane and the dura were closed in an ordinary fashion.

### 4:18 Postoperative Imaging and Postoperative Course of Treatment.

Early postoperative MRI proved gross-total resection of the tumor. Pathology analysis revealed choroid plexus papilloma grade I. Postoperative CT reconstruction showed the craniotomy sized 3.6 by 2.6 cm. In the postoperative period, the patient did not have any CSF leak, additional neurological disorders, or any other complications.

### 4:42 Conclusions.

The exoscope proved to be a useful instrument that facilitated visualization of the lesion of any part of the fourth ventricle, even the aqueduct and the ventricular roof, which allowed minimizing the size of dissection and performing the access to the tumor via minimal possible approach. It also ensured a comfortable positioning of the surgeon during all steps of the procedure. The use of midline suboccipital osteoplastic craniotomy reduced potential risks of complications, in particular, CSF leak and craniovertebral junction instability. These advantages contributed to minimal damage to surrounding tissues, high precision of manipulations, and consequently a successful outcome for the patient.

## Acknowledgments

We thank Ms. Aliya Bukhtoyarova for language editing and narration.

## Disclosures

The authors report no conflict of interest concerning the materials or methods used in this study or the findings specified in this publication.

## Author Contributions

Primary surgeon: AA Sufianov, RA Sufianov. Assistant surgeon: Iakimov. Editing and drafting the video and abstract: RA Sufianov, Gaysin. Critically revising the work: AA Sufianov, RA Sufianov. Reviewed submitted version of the work: AA Sufianov, RA Sufianov, Gaysin. Approved the final version of the work on behalf of all authors: AA Sufianov. Supervision: AA Sufianov, RA Sufianov.

## Supplemental Information

### Patient Informed Consent

The necessary patient informed consent was obtained in this study.
